# Hippocampal atrophy, asymmetry, and cognition in type 2 diabetes mellitus

**DOI:** 10.1002/brb3.741

**Published:** 2017-12-22

**Authors:** Nicole T. Milne, Romola S. Bucks, Wendy A. Davis, Timothy M. E. Davis, Ronald Pierson, Sergio E. Starkstein, David G. Bruce

**Affiliations:** ^1^ School of Psychology University of Western Australia Western Australia Australia; ^2^ School of Medicine & Pharmacology University of Western Australia Western Australia Australia; ^3^ Brain Image Analysis Technology Innovation Center Coralville IA USA; ^4^ School of Psychiatry & Clinical Neuroscience University of Western Australia Western Australia Australia

**Keywords:** cognition, hippocampus, magnetic resonance imaging, type 2 diabetes

## Abstract

**Introduction:**

Type 2 diabetes mellitus is associated with global and hippocampal atrophy and cognitive deficits, and some studies suggest that the right hippocampus may display greater vulnerability than the left.

**Methods:**

Hippocampal volumes, the hippocampal asymmetry index, and cognitive functioning were assessed in 120 nondemented adults with long duration type 2 diabetes.

**Results:**

The majority of the sample displayed left greater than right hippocampal asymmetry (which is the reverse of the expected direction seen with normal aging). After adjustment for age, sex, and IQ, right (but not left) hippocampal volumes were negatively associated with memory, executive function, and semantic fluency. These associations were stronger with the hippocampal asymmetry index and remained significant for memory and executive function after additional adjustment for global brain atrophy.

**Conclusions:**

We conclude that asymmetric hippocampal atrophy may occur in type 2 diabetes, with greater atrophy occurring in the right than the left hippocampus, and that this may contribute to cognitive impairment in this disorder. These cross‐sectional associations require further verification but may provide clues into the pathogenesis of cognitive disorders in type 2 diabetes.

## 1 INTRODUCTION

Type 2 diabetes mellitus (T2DM) is associated with widespread cognitive decrements (Palta, Schneider, Biessels, Touradji, & Hill‐Briggs, 2014). In later life, there is also an increased risk of dementia, Alzheimer's disease, and vascular dementia (Biessels, Staekenborg, Brunner, Brayne, & Scheltens, 2006; Cheng, Huang, Deng, & Wang, 2012). While the mechanisms linking diabetes, cognitive decrements, and dementia are incompletely understood, they are most likely multifactorial and both neurodegenerative and cerebrovascular changes may play a role.Structural brain abnormalities associated with T2DM include reductions in total brain volumes, gray matter volume loss in cortical regions, and increased white matter lesions in periventricular and subcortical regions (Brands et al., 2007). Hippocampal atrophy has also been reported in T2DM (Gold et al., 2007; Hayashi et al., 2011; Kamiyama et al., 2010) and accelerated loss of hippocampal volume in a longitudinal study (Debette et al., 2011). Reduced hippocampal volume has been linked to diabetes‐related cognitive dysfunction (Hayashi et al., 2011; Moran et al., 2013), and gray matter atrophy, including the hippocampus, explained a substantial amount of cognitive decrements due to T2DM in one of these studies (Moran et al., 2013).

In healthy adults, the right hippocampus is reliably larger than the left (R > L asymmetry) (Pedraza, Bowers, & Gilmore, 2004). This direction of asymmetry is generally maintained in preclinical and clinical Alzheimer's disease, where hippocampal atrophy is an early phenomenon (Shi, Liu, Zhou, Yu, & Jiang, 2009). This maintenance of the usual R > L direction of asymmetry in Alzheimer's disease could occur because of equal rates of atrophy in both structures or possibly increased atrophy of the left hippocampus (Barnes et al., 2005; Cherbuin, Réglade‐Meslin, Kumar, Sachdev, & Anstey, 2010; Shi et al., 2009). Whether the direction of asymmetry follows the same pattern in T2DM as in Alzheimer's disease is unknown but could provide useful information on the pathogenesis of dementia in diabetes. The aim of this study was to examine left and right hippocampal volumes, and determine the direction and magnitude of hippocampal asymmetry, in relation to cognitive functioning in older, dementia‐free adults with T2DM.

## 2 MATERIAL AND METHODS

The participants in this study were recruited to a longitudinal study of cognition in T2DM, and were a subgroup of participants in the Fremantle Diabetes Study Phase II (FDS2; Davis, Bruce, & Davis, 2012), a longitudinal, observational study of known diabetes being conducted in the port city of Fremantle, Western Australia. Details of recruitment procedures to the FDS2 and sample characteristics, including classification of diabetes type and nonrecruited persons with diabetes, have been published previously (Davis et al., 2012). In the present study, consecutive FDS2 recruits were invited to participate in an additional cognition/imaging study and recruitment to this study continued until the planned study sample (*n *=* *120 with completed cognitive assessments/magnetic resonance imaging [MRI] studies) which met the inclusion/exclusion criteria was achieved. The inclusion criteria for this study included age ≥60 years, diabetes of at least 10 years duration, absence of dementia, and having English language proficiency for cognitive testing. The exclusion criteria included severe medical conditions (severe organ dysfunction, cancer), contraindications for magnetic resonance imaging (metal implants, pacemaker, claustrophobia), or neurological/psychiatric diagnoses. The sample size was based on published rates of cognitive decline in type 2 diabetes (Bruce et al., 2008) and estimated to be powered to detect associations with global brain volume changes in a 3‐year follow‐up study. The study was approved by the Human Research Ethics Committees of the Southern Metropolitan Area Health Service and the University of Western Australia. Written informed consent was obtained from all participants.

### 2.1 Magnetic resonance imaging

MRI data were acquired at Fremantle Hospital on a 1.5 Tesla Siemens Avanto system using a research protocol. The imaging protocol included the following sequences: (i) Multiplanar “FLASH” localizer, (ii) Coronal 3D T1‐weighted echo orthogonal to the AC‐PC line, (iii) Coronal proton density and T2‐weighted fast spin echo oriented parallel to 2. Intracranial, total brain, hippocampal, and amygdalar volumes were extracted using a semiautomated system (BRAINS‐2 AutoWorkup function (RRID:SCR_006618); Pierson et al., 2011). Hippocampal and amygdala volumes were extracted from the Regions of Interest (ROI's) created from the Automated Neural Network program embedded in BRAINS‐2. All scans were manually checked and traced where required. Total brain, hippocampal, and amygdalar volumes were transformed to percentage of intracranial volume prior to analyses. For each participant, a Hippocampal Asymmetry Index (HAI) score was derived from raw hippocampal volumes (HcVs), using the formula (adapted from Cherbuin et al., 2010, by reversing the numerator for ease of interpretation): HAI = ([Right HcV – Left HcV]/Total HcV) × 100. The theoretical range of the HAI is −100 to 100, with positive scores indicating R > L hippocampal volume and higher absolute values representing greater magnitude of asymmetry. Participants were allocated to one of three groups based on their HAI (L > R, L = R, R > L), and those with HAI scores between −1.0 and +1.0 were classified as symmetric (L = R). Amygdalar volumes were treated in a similar fashion, including constructing an Amygdalar Asymmetry Index as a control brain structure.

### 2.2 Cognitive measures

Global cognition was assessed with the Mini Mental State Examination (MMSE) (Folstein, Folstein, & McHugh, 1973) and the Clinical Dementia Rating (CDR) scale (Morris, 1993). With the CDR, a trained researcher collects data from both the participant and a suitable informant and rates six domains: memory, orientation, judgment/problem solving, community affairs, function at home/hobbies, and personal care. These are then used to generate ratings of normal cognition (CDR 0) or severity levels of dementia (CDR 1–3) and an intermediate category (CDR 0.5) defines cognitive impairment of mild degree without functional impairment. In this study, the inclusion criteria permitted individuals with CDR 0 and 0.5.

A comprehensive cognitive test battery was administered in a fixed order by a trained researcher during a single ~90 min session. The Cognitive Drug Research System (CDRS; Keith, STANISLAV, & Wesnes, 1998) computerized test battery was used to measure speed and accuracy of performance during memory and attention subtests, followed by the Trail Making Test (TMT; Reitan, 1955), CLOX clock‐drawing (Royall, Cordes, & Polk, 1998), and verbal fluency (“FAS” and “Animals”) as measures of executive function and semantic fluency. The National Adult Reading Test, Second edition (Nelson & Willison, 1991) was used to estimate pre‐morbid, WAIS‐R full scale IQ (FSIQ). Subtest measures from the CDRS were collapsed into the five cognitive factor scores described by Wesnes, Ward, McGinty, and Petrini (2000). (i) *Power of Attention* was obtained from CDRS measures of simple reaction time (RT), choice RT, and digit vigilance subtests: the combined total is reported in milliseconds, with higher scores representing slower performance. (ii) *Continuity of Attention* represents attentional accuracy and errors (false alarms) during CDRS digit vigilance and choice RT subtests; 100% accuracy produces a maximum score of 95. (iii) *Quality of Working Memory* assesses working memory substrates, the visuospatial sketchpad and phonological loop, via target identification and discrimination on CDRS spatial and numeric working memory tasks; 100% accuracy corresponds to a maximum score of 2. (iv) *Quality of Episodic Memory* is a combined measure of visual and verbal memory accuracy on CDRS immediate and delayed word recall, delayed word recognition, and delayed picture recognition subtests. Percentage accuracy on each subtest is summed, producing a potential maximum of 400. (v) *Speed of Memory* was derived from CDRS measures of RT on the two working memory (spatial and numeric) and two delayed recognition (word and picture) memory subtests; the combined total is reported in milliseconds, with higher scores representing slower performance. (vi) An *Executive Function* score combined measures of generativity (letter fluency, “FAS” total words), conceptual switching (TMT B/A ratio score), and planning/organization (CLOX2 – CLOX1 score). Subtest scores were standardized using age‐matched normative data (TMT, Tombaugh, 2003; FAS, Tombaugh, Kozak, & Rees, 1999; CLOX, RS Bucks & M Weinborn, personal communication, 2013), and the composite Executive Function factor was calculated as the mean of the *z* scores for the three subtests. (vii) *Semantic Fluency* was assessed with the category (“Animals”) fluency test and standardized against normative data (Tombaugh et al., 1999).

### 2.3 Statistical analyses

All statistical analyses were performed using IBM SPSS Version 21 (IBM Corp., Armonk, NY) (RRID:SCR_002865). Hippocampal asymmetry groups (L > R, L = R, and R > L) were compared on MRI‐derived measures of total brain, and separate left and right hippocampal volumes, using analysis of variance. Relationships between MRI‐derived brain measures and cognitive factors were examined using partial correlations controlling for age, sex, and estimated FSIQ. Given non‐normality in the distributions of some cognitive variables, bootstrap sampling (*B *=* *1000) was applied and bias‐corrected and accelerated confidence intervals are reported.

## 3 RESULTS

### 3.1 Sample characteristics

The participant characteristics are summarized in Table 1. All participants were community dwelling with a mean age of 73.5 ± 7.0 years, 67.5% (81/120) were male and the mean duration of diabetes was 21.2 ± 5 years. The majority of the sample (71.6%, 86/120) were rated as having normal cognition (CDR 0) and the remainder were rated as CDR 0.5.

**Table 1:** Demographic, imaging and cognitive characteristics of 120 study participants with type 2 diabetes

Characteristic	
Sex, male *n (%)*	81 (67.5)
Age, years	73.5 ± 7.0
Hand, right *n (%)*	117 (97.5)
Education, years	11.4 ± 3.1
Estimated full scale IQ	104 ± 11.0
Mini Mental State Examination score	28.3 ± 1.7
Clinical Dementia Rating, 0/0.5 *n (%)*	86/34 (71.6/28.3)
Intracranial volume *cm* ^*3*^	1410.16 ± 132.78
Total brain volume *cm* ^*3*^	1097.60 ± 95.72
Hippocampal volume *cm* ^*3*^:	
Total	3.70 ± 0.56
Left	1.87 ± 0.27
Right	1.83 ± 0.31
Hippocampal Asymmetry Index *%*	−1.38 ± 4.99
Amygdala volume *cm* ^*3*^:	
Total	2.17 ± 0.27
Left	1.07 ± 0.15
Right	1.10 ± 0.15
Amygdalar Asymmetry Index *%*	1.62 ± 5.48
Cognitive factors:	
Power of attention *(ms)*	1357.44 ± 190.53
Continuity of Attention	87.93 ± 5.61
Quality of Episodic Memory	159.34 ± 48.00
Quality of Working Memory	1.70 ± 0.36
Speed of Memory *(ms)*	4940.33 ± 1162.73
Executive Function *z score*	−0.50 ± 0.92
Semantic Fluency *z score*	−0.25 ± 1.08

Data are presented as mean ± *SD* unless otherwise stated.

### 3.2 Hippocampal asymmetry

**Figure 1:**
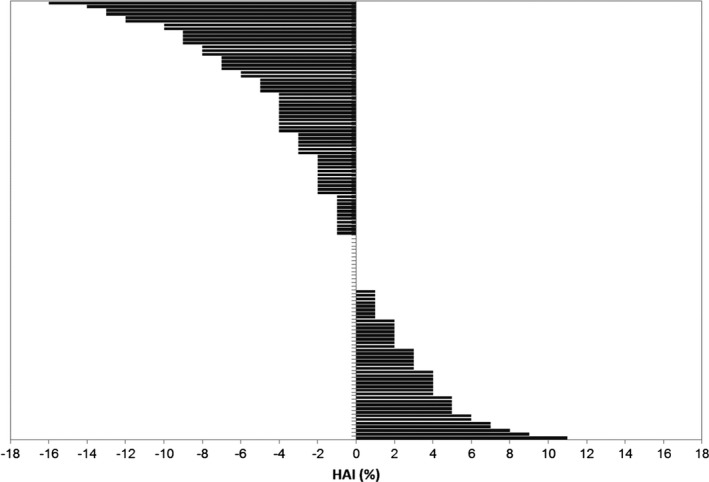
Hippocampal Asymmetry Index (HAI) in 120 participants with type 2 diabetes. Each bar represents one case. Negative values indicate left greater than right hippocampal asymmetry and positive values, the opposite

The mean HAI score was slightly leftward (*M *=* *−1.38, *SD* = 4.99), and the majority of participants (49.2%, 59/120) had asymmetry with a L > R direction, 30% (36/120) had asymmetry with a R > L direction and 20.8% (25/120) had equal left and right volumes (Figure 1). The three HAI groups were investigated as demonstrated in Table 2 and no differences were seen in age, sex, handedness, total brain and left hippocampal volumes. There was a significant difference in right hippocampal volumes, *F*(2,117) = 8.3218, *p *<* *.001, η^2 ^= 0.12, and the participants with L > R HAI had significantly smaller right hippocampal volumes compared with those with R > L HAI, (mean difference = −0.02, *p *<* *.001) and compared with those with symmetric HAI (mean difference = −0.01, *p *=* *.03).

**Table 2:** Demographic, total brain and hippocampal volumes by three Hippocampal Asymmetry Index (HAI) groups (L > R indicates leftward asymmetry, L = R indicates symmetry, R > L indicates rightward asymmetry)

	Hippocampal Asymmetry Index groupings			
	L > R *n *=* *59	L = R *n *=* *25	R > L *n *=* *36	*p*‐value
Age, years	73.3 ± 6.4	72.6 ± 8.6	74.4 ± 6.7	.60
Sex (% male)	76.3	60.0	58.3	.13
Handedness (% left)	3.4	0	2.8	.55
Clinical dementia rating (% 0.5)	28.8	32.0	25.0	.83
Lt hippocampus (% ICV)	0.134 ± 0.022	0.137 ± 0.026	0.131 ± 0.019	.57
Rt hippocampus (% ICV)	0.122 ± 0.026	0.137 ± 0.026	0.142 ± 0.02	<.01
Total brain (% ICV)	77.34 ± 4.00	77.96 ± 5.08	79.13 ± 4.06	.14
Intracranial volume (cm^3^)	1428.19 ± 122.68	1423.08 ± 156.41	1371.63 ± 126.61	.11

Data are presented as mean ± *SD* unless otherwise stated.

To assess whether the direction of asymmetry was specific to the hippocampus, amygdalar volumes were used for comparison. The amygdalar asymmetry index was slightly rightward (*M *=* *1.48, *SD* = 5.69) and there was no correlation between amygdalar and hippocampal asymmetry indices (*r *=* *.11, *p *=* *.26) after controlling for age and sex.

### 3.3 Hippocampal measures and cognition

Table 3 presents the results of partial correlations between the neuroimaging and cognitive variables. The left hippocampal volume was negatively associated with attention speed (Power of Attention) only. In contrast, right hippocampal volumes and greater leftward asymmetry (L > R HAI) were each negatively associated with attention speed, Quality of Episodic Memory, Executive Function, and Semantic Fluency. In addition, right hippocampal volume was also negatively associated with Speed of Memory, that is, slower memory retrieval.

**Table 3:** The association between MRI brain measures and cognitive performance in Type 2 diabetes

	Hippocampal volume^a^							
	Left		Right		Hippocampal asymmetry		Total brain volume^a^	
Cognitive domain	Correlation [95% CI]	*p*	Correlation [95% CI]	*p*	Correlation [95% CI]	*p*	Correlation [95% CI]	*p*
Power of attention (ms)^b^	−0.21 [−0.37, −0.05]	.023	−0.30 [−0.47, −0.14]	.001	−0.23 [−0.40, −0.07]	.014	−0.31 [−0.42, −0.18]	.001
Continuity of attention	0 [−0.12, 0.13]	.99	0.01 [−0.13, 0.15]	.91	−0.01 [−0.18, 0.17]	.95	−0.02 [−0.18,0.04]	.82
Quality of episodic memory	0.06 [−0.11, 0.23]	.53	0.21 [0.02, 0.38]	.027	0.26 [0.08, 0.43]	.005	0.18 [−0.00, 0.35]	.054
Quality of working memory	0.06 [−0.10, 0.22]	.52	0.13 [−0.04, 0.31]	.15	0.16 [−0.02, 0.32]	.10	0.18 [0.03, 0.33]	.048
Speed of memory (ms)^b^	−0.18 [−0.33, −0.02]	.06	−0.23 [−0.37, −0.09]	.014	−0.15 [−0.32, 0.02]	.10	−0.31 [−0.44, −0.16]	.001
Executive function	0.11 [−0.06, 0.27]	.25	0.24 [0.05, 0.40]	.010	0.28 [0.07, 0.49]	.002	0.10 [−0.06, 0.28]	.31
Semantic fluency	0.12 [−0.02, 0.26]	.20	0.20 [0.07, 0.33]	.030	0.21 [0.02, 0.37]	.027	0.18 [0.03, 0.33]	.06
Significant results, controlled for total brain volume:								
Quality of episodic memory					0.23 [0.07, 0.40]	.013		
Executive function			0.22 [0.02, 0.41]	.017	0.27 [0.04, 0.46]	.004		

Partial correlations controlled for sex, age, and estimated FSIQ.

a Adjusted for intracranial volume.

b Higher scores indicate poorer performance.

There were significant associations between Power of Attention, Quality of Working Memory, Speed of Memory, and total brain volume (all *p *<* *.05). To account for the possible influence of global atrophy, the analyses were repeated with total brain volume added as a covariate. In this analysis, greater leftward hippocampal asymmetry remained negatively associated with Quality of Episodic Memory and Executive Function (all *p *<* *.02) and right hippocampal volume remained negatively associated with Executive Function (*p *=* *.017).

## 4 DISCUSSION

This study aimed to investigate right and left hippocampal volumes individually, hippocampal asymmetry, and their relationship with cognitive performance in older adults with type 2 diabetes. In contrast to the right‐greater‐than‐left hippocampal asymmetry usually seen in healthy adults (Pedraza et al., 2004) and in Alzheimer's disease (Shi et al., 2009), the majority of our participants exhibited a reversal of direction, displaying larger left than right hippocampal volumes. Specifically, a hippocampal asymmetry index of −1.38 was obtained in this study, whereas a recent comparable study obtained an index of 3.91 in a healthy older sample (Cherbuin et al., 2010). The direction and magnitude of hippocampal asymmetry was associated with performance across multiple cognitive domains including memory, executive function, semantic fluency, and processing speed. Atrophy of the right hippocampus rather than changes on the left appeared to explain the anatomical asymmetry and the cognitive associations. These associations remained significant for episodic memory and executive function, cognitive domains commonly compromised in type 2 diabetes (Palta et al., 2014), after controlling for global atrophy also associated with this condition (Brands et al., 2007). We conclude that asymmetric hippocampal atrophy, with greater atrophy occurring in the right hippocampus, contributes to cognitive decrements in type 2 diabetes. Right hippocampal atrophy could be a hitherto unknown characteristic that could assist with the study of cognitive disorders in type 2 diabetes.

Previous studies have demonstrated hippocampal atrophy in type 2 diabetes (Debette et al., 2011; Gold et al., 2007; Hayashi et al., 2011; den Heijer et al., 2003; Kamiyama et al., 2010; Moran et al., 2013), but few specifically explored laterality. Several previous findings are consistent with this study. Type 2 diabetes has been associated with smaller right temporal volumes (Chen, Li, Sun, & Ma, 2012) and with cortical volume loss in the right hemisphere (Brundel et al., 2010). In a study of nondiabetic women at risk for Alzheimer's disease, an inverse relationship between insulin resistance and right hippocampal volumes was seen (Rasgon et al., 2011).

A number of other conditions may exert a greater effect on the right hippocampus than on the left. These include carriage of the apolipoprotein E ɛ4 allele (Geroldi et al., 2000) and mood disorders, including posttraumatic stress disorder (Pavic et al., 2007), bipolar disorder (Haukvik et al., 2014), major depression (Mathias et al., 2016), and anxiety (Levita et al., 2014). A possible common factor shared with these conditions and with type 2 diabetes is dysfunction of the hypothalamic‐pituitary‐adrenal axis (Bruehl et al., 2007) suggesting chronic stress associated with diabetes as a common mechanism.

The study has several limitations. The associations are cross‐sectional only preventing assessments of the direction of causality, although the long duration diabetes in the participants makes reverse causation less likely. The study was conducted only in diabetes, without nondiabetic controls, necessitating comparisons from the literature. In addition, the study has relatively low power given the limited sample size. Longitudinal studies are indicated to confirm the study findings and to determine whether they can predict cognitive decline in type 2 diabetes. The study strengths include the source of the study sample, the comprehensive nature of the cognitive assessment that included clinical and neuropsychological assessments and the MRI procedure that included advanced, automated volume measures that help avoid biased assessments (Maltbie et al., 2012).

In conclusion, the findings of the present project suggest that some of the cognitive decrements associated with type 2 diabetes are associated with right rather than left hippocampal atrophy. The reasons for this apparent vulnerability are unknown and further studies are indicated given their potential utility in unraveling the cause of cognitive decline in type 2 diabetes.
